# *Rhizobium* and Mycorrhizal Fungal Species Improved Soybean Yield Under Drought Stress Conditions

**DOI:** 10.1007/s00284-021-02432-w

**Published:** 2021-03-09

**Authors:** Ozede N. Igiehon, Olubukola O. Babalola

**Affiliations:** Food Security and Safety Niche, Faculty of Natural and Agricultural Science, North-West University, Private Mail Bag X2046, Mmabatho, 2735 South Africa

## Abstract

Food insecurity is a serious threat due to the increasing human population particularly in developing countries and may be minimized by the use of microbial inoculants. Also, the problems of excessive use of chemical fertilizers including the fact that most of the fertilizers are relatively non-affordable and that they also contaminate underground and surface water, which can increase the risk of blue baby syndrome in infants and stomach cancer in adults. There is therefore the need to harness a more cost-effective, eco-friendly and beneficial biological agents to improve crops productivity especially under drought conditions. Thus, in this study, the ability of rhizobia species and arbuscular mycorrhizal fungi (AMF) to enhance soybean tolerance to drought stress under water regimens of 100, 70 and 40% field capacity (FC) was investigated. It was observed that co-inoculation of soybean with *Rhizobium* spp. (R1+R3) as well as with *Rhizobium* spp. and mycorrhizal consortium (R1+R3MY) had significant impacts (*P* < 0.05) on soybean leaf relative water content and electrolyte leakage, respectively. The levels of proline increased mainly in microbially amended soybean exposed to drought stress. Plants inoculated with R1+R3MY showed the highest number of spore and % mycorrhization in all the water regimes. At 40% FC, R1+R3MY treatment was found to promote soybean growth compared to the non-inoculated plants. Similarly, at 40% FC, R1+R3MY inoculum had the greatest impacts on soybean pod number, seed number, seed fresh weight, highest seed number per pod and seed dry weight while at 70% water stress, significant impacts of R1MY inoculation were observed on pod number, pod fresh weight and seed dry weight. These results revealed that co-inoculation of rhizobia and mycorrhizal fungi can be harnessed biotechnologically to proffer solution to food insecurity.

## Introduction

Drought stress is one of the most damaging abiotic factors affecting global food security. Drought stress might range from moderate and short to very severe and protracted duration, limiting crop yield [1]. This abiotic stress is estimated to cause severe growth problems in plants for over 50% of the arable lands by the end of the next three decades [2–4]. For instance, drought interfere with plant normal functions by affecting its water potential and turgor leading to changes in physiological and morphological parameters [5, 6]. Growth parameters under drought stress have been investigated in many crops such as barley, maize, wheat, rice and soybean [7, 8]. Water content and fresh weight are among the common growth parameters that are influenced by drought stress [9]. Another problem of drought stress is that it influences the availability and movement of nutrients, since soil nutrients are transported to the roots by water. Drought therefore reduces nutrient diffusion as well as mass flow of soluble nutrients such as Si, Ca, Mg, sulfate and nitrate [10, 11] and these problems can be surmounted through the use of rhizobia and mycorrhizal fungal species.

Furthermore, several studies involving the use of valuable soil microorganisms (e.g. rhizobia) to reduce drought stress have been investigated [12]. Other studies have demonstrated that inoculation with AMF enhanced water absorption in numerous plants that were subjected to drought stress [13, 14]. Specifically, under harsh conditions, AMF mycelia can penetrate larger volume of soil than host plant roots, therefore increasing water absorption and transport to the root and other plant parts which eventually improves the osmotic regulation, cellular and physiological effects in plants [15].

Co-inoculation of soybean plants with AM fungal and *Rhizobium* species enhanced nodule biomass in a semi-arid environment [16]. Similarly, co-inoculation of *Glomus* spp. with *Rhizobium leguminosarum* significantly enhanced plant biomass and other plant parameters of *Pisum sativum* [17] while in another study, dual inoculation of *Rhizobium* spp. and mycorrhizal consortium reduced drought stress and enhanced shoot relative water content, fat content and yield of soybean plants [16]. In addition, studies have also shown the effectiveness of co-inoculation of specific rhizobia and mycorrhizal fungi in a controlled environment [18, 19] but more work with different rhizobia and mycorrhizal combinations is needed in this aspect. However, single inoculation of *Rhizobium* spp. improved soybean seed germination under drought stress imposed by 4% poly-ethylene glycol under a controlled environment [8]. Inoculation of pea with *Variovorax paradoxus* 5C-2 under drought stress resulted in higher seed yield, seed N accumulation, seed number and nodulation restoration [20]. Bacteria with 1-aminocyclopropane-1-carboxylate deaminase (ACC) deaminase producing potential reduced the effects of drought on growth, yield and ripening of pea under controlled and field conditions [21] while inoculation of *Pisum sativum* with *Pseudomonas fluorescens* biotype G (ACC-5) under arid condition increased root length and water uptake from soil. Increased nodulation by N-fixing bacterial symbionts eliminated drought-induced reduction in nodulation and seed N content. Co-inoculation of a leguminous plant *Cicer arietinum* with *Mesorhizobium ciceris*, *Pseudomonas* sp. and *Bacillus* sp. significantly enhanced seed germination, shoot and root length of the plant over the non-inoculated control [22].

Thus, the aim of this study is to determine the effects of single and co-inoculation of rhizobia and AMF on soybean growth and productivity exposed to drought stress under a controlled environment with the prospect of harnessing the effective microbial inoculants for field application.

## Materials and Methods

### Experimental and Soil Collection Sites

Soil samples for pot experiment were collected from North-West University farm while the greenhouse experiment was also conducted in North-West University, South Africa.

### Experimental Set-Up and Soybean Growth Conditions

Soil used for the greenhouse experiment was homogenized and sieved using a 5.6 mm diameter round sieve. Sieved soil samples were packed in autoclavable plastics and sterilized at 121 °C for 30 min in an autoclave (SA-300VL Autoclave, Taiwan.

The experimental trial was set-up using 3 factorial completely randomized design (CRD) and the three factors included rhizobia, arbuscular mycorrhizal fungi (AMF) consortium and drought or water stress. ‘The experiment was conducted in a greenhouse under natural light’.

Three drought regimes were used in this study namely: 100, 70 and 40% field capacity (FC). The water stress levels (70 and 40% FC) were determined by the method described by Zarik et al. [23] with little modifications. Six (6) small plastic pots were weighed (W1), the plastic pots + 100 g of soil were weighed (W2) and weight of 100 g of soil only (W3) was obtained by subtracting W2 from W1. The first plastic pot with soil was watered with 40 ml of water until ‘soil saturation’, the second, third, fourth, fifth and sixth pots were watered with 30, 20, 15 and 10 and 5 ml of water, respectively. Watered pots were sealed with plastic and kept on the lab shelf. The sixth pot watered with 5 ml of water was not waterlogged after observing for 3 days and was weighed again to obtain its saturated weight and water holding capacity of the soil was subsequently calculated from this pot using the formula:

$$\rm {Water holding capacity }= \frac{\rm {Saurated weight}-\rm {dry weight}}{\rm {dry weight }}\times 100\rm {\%}$$

Then, the water holding capacity (which represents the amount of water needed to saturate the soil) of the 8 kg of soil (used for the greenhouse experiment) was obtained from the water holding capacity obtained for the 6th pot above by direct proportionality.

The two rhizobia *Rhizobium* sp. strain R1 with the accession no. MG309875 (https://www.ncbi.nlm.nih.gov/search/all/?term=.%20MG309875%20) and *Rhizobium cellulosilyticum* strain R3 with the accession no. MG309874 (https://www.ncbi.nlm.nih.gov/search/all/?term=MG309874) used in this study were isolated in our previous work from Bambara groundnut rhizosphere and identified by molecular methods. In the previous work, these bacteria were also found to possess some plant growth promoting traits and were tolerant to in vitro osmotic stress induced by stress-stimulant [polyethylene glycol (PEG)] while the AMF consortium [consisted of *Paraglomus occulum* (molecular determination), *Gigaspora gigantea*, *Funneliformis mosseae* (previously *Glomus mosseae*), *Claroideoglomus etunicatum* (previously *Glomus etunicatum*) and *Rhizophagus clarus* (previously *Glomus clarum*)] was obtained from Department of Biochemistry and Microbiology, Rhodes University, Grahamstown, South Africa.

*Rhizobium* sp. strain R1 and *R. cellulosilyticum* strain R3 were grown and harvested according to the method of Prakamhang et al. [24] with little modifications. Fresh cultures of the bacteria were grown in flasks containing 1000 ml nutrient broth in a shaker incubator (FMH200 Instruments) at 180 rpm for 6 days. Fully grown culture was centrifuged at 1000×*g* for 10 min and washed twice in a sterile 0.85% saline solution. The initial optical density (OD) of the bacteria was adjusted to 1.3 OD and the bacterial titer was adjusted to 30 × 10^9^ and 31 × 10^9^ CFU (colony forming unit) ml^−1^ for *Rhizobium* sp. strain R1 and *R. cellulosilyticum* strain R3, respectively.

The greenhouse experiment was conducted in plastic pots (30 cm diameter, height 29 cm) containing 8 kg of dried and sterile soil. The experiment which had a total number of 192 replicates comprised 24 treatments and each treatment had 8 replicates. Soybean seeds (PAN 1532 R cultivar) were washed in sterile distilled water, surface sterilized in 75% ethanol and then 1% sodium hypochlorite and rinsed severally to totally remove the chemicals. For single rhizobial inoculation, approximately 240 surface sterilized soybean seeds were inoculated with 200 ml of *Rhizobium* sp. strain R1and *R. cellulosilyticum* strain R3 suspension each, co-inoculation was done by amending approximately 240 surface sterilized soybean seeds with 100 ml of *Rhizobium* sp. strain R1 and *R. cellulosilyticum* strain R3 suspension each and 200 ml of sterile 0.85% saline water was added to approximately 480 surface sterilized soybean seeds for AMF consortium and control treatments. Flasks containing seeds were agitated (to thoroughly mix the seeds with the inocula) in a shaker incubator at 180 rpm at 28±2°C for 24 hr. Thereafter, the liquid suspension was decanted and air-dried on a sterile aluminum foil paper in a sterilized laminar flow cabinet (Filta Matix Laminar Flow Carbinet) prior to sowing. All the greenhouse pots containing 8 kg of sterile soil were watered with 360 ml of water (100% FC) before sowing. Five seeds were sowed per pot. Treatments involving AM fungi were inoculated by placing a tea spoonful (approximately 5 g) of AMF consortium in the soil before adding the seeds. Soybean bean plants were thinned to two plants per pot two weeks after emergence. One of the plants was marked and termed as ‘plant for sample collection (PSC)’ used for assessment of physiological, biochemical and % mycorrhization parameters while the second plant was allowed to grow to maturity which was used to assessed the below-ground-above-ground and/or morphological parameters. All the water treatments (100, 70 and 40% FC) were fully watered to the field capacity after every 72 h for approximately three weeks after germination and thereafter, drought or water stress was initiated in the 70 and 40% FC treatments and were watered after every 48 h with 252 and 144 ml of water respectively but we continued watering the 100 % FC treatments (the watering control) with 360 ml of water. Three weeks after initiating drought stress, pots were watered after every 72 h until termination of the experiment so as to increase drought stress.

### Physicochemical Analysis of Soil

The physicochemical analysis of the soil for the greenhouse experiment was determined following previously reported methods [16].

### Parameters Measured

#### Relative Water Content

The leaf relative water content was determined according to the methods described by Aroca et al. [25] with little modifications and the ‘youngest fully developed leaves of each plant’ were used. Fresh leaf samples were weighed (Fresh weight-FW) and placed in test tubes saturated with water and kept at 4 °C for 48 h. Thereafter, the leaf samples were weighed again to obtain the turgid weight (TW) and oven**-**dried at 60 °C for 24 h and dry weights (DW) were obtained. The leaf relative water content was calculated as follows:

$$\rm {Leaf relative water content }(\rm {\%}) =\frac{(\rm {FW}-\rm {DW})}{(\rm {TW}-\rm {DW})}\times 100$$

where FW—fresh weight, DW—dry weight and TW—turgid weight.

#### Electrolyte Leakage

The youngest leaves of approximately the same size from the youngest branch ‘toward the distal end’ were collected from the sampling plants and thoroughly rinsed with de-ionized water to remove electrolytes attached to the leaf surfaces. Leaf samples were placed in 45 ml falcon tubes containing 10 ml deionized of H_2_O and incubated on a shaker incubator at 28 ± 2°C for 24 h. Subsequently, electrical conductivity of the liquid suspension (Lt) was obtained using conductivity meter (PL-700AL, Taiwan) and samples were autoclaved at 120 °C for 1200 s, cooled to 25 °C and electrical conductivity of the liquid (L0) was obtained. The electrolyte leakage was calculated as follows [26]:

$$\rm {Electrolyte leakage }(\rm {\%})=\frac{\rm {Lt}}{\rm {L}0}\times 100\rm {\%}$$

where Lt—electrical conductivity of the liquid suspension prior to autoclaving and L_0_—electrical conductivity of the liquid suspension after autoclaving.

#### Determination of Leaf Proline Content

Proline concentration was determined according the methods of Ortiz et al. [26] with little modifications. Briefly, 1.25 g of ninhydrin was dissolved in 20 ml of 6 M phosphoric acid and 30 ml of glacial acetic acid by heating on a hot-plate with agitation. The solution was allowed to cool and kept at 4 °C and the solution became stable after 24 h. Approximately 500 mg of fresh soybean leaf sample was ground in 10 ml of 3% aqueous sulfo-salicyclic acid and centrifuged at 10000×*g* for 10 min. Two ml (2 ml) of the supernatant was reacted with 2 ml of glacial acetic acid and 2 ml of acid-ninhydrin solution in 45 ml falcon tubes at 100 °C in a water bath for 60 min and the reaction was stopped in an ice box. Four ml (4 ml) of toluene was added to extract the mixture and agitated vigorously for 15–20 s in a shaker incubator at 250 rpm. The mixture was kept in the dark for 30 min and the ‘chromophore containing toluene was aspirated from the aqueous phase and the absorbance was read at 520 nm using toluene for a blank’. The concentration of proline was estimated from a standard curve ‘established with a reference proline solution’. Briefly, 1 mg/ml stock solution of proline was prepared by weighing 10 mg of proline (DL-Proline, China) in 10 ml of sterile water. 0, 50, 100, 150, 200, 250, 300 µl of the stock solution was pipetted into seven tubes containing 300, 250, 200, 150, 100, 50 and 0 µl of sterile water, respectively. The mixtures were then reacted with 2 ml of glacial acetic acid and 2 ml of acid-ninhydrin solution in 45 ml falcon tubes at 100 °C in a water bath for 60 min and the reaction was stopped in an ice box. The mixture was vigorously agitated using a vortex (Vortex Genie, U.S.A) after adding 4 ml of toluene. The mixture was kept in the dark for 30 min and the absorbance of the proline-containing upper layer ‘was read at 520 nm using toluene for a blank’ and proline standard curve was plotted from the absorbance values.

#### Chlorophyll Content

Chlorophyll content was taken from the youngest fully developed leaf at the distal end of PSC using a chlorophyll content meter (CCM-200 plus).

#### AMF Spore Estimation

AM fungal spores were estimated by wet sieving and decanting methods as described by Pacioni [27] with little modifications. Fifty g (50 g) of soil close to soybean root region was collected and mixed in 500 ml of sterile distilled water. The mixture was passed through a series of sieves of different sizes stacked together in an increasing order from the base to the top: 53, 63, 106 and 212 µm. The trapped fungal spores on the 53 µm were rinsed into a 1.5 ml vials and centrifuged at 1800 rpm for 5 min. The supernatant (1 ml) was mixed with 60% sucrose (0.5 ml) in a 1.5 ml vial and centrifuged at 1800 rpm for 5 min. The supernatant**-**containing AMF spores was decanted in a cleaned Petri-dish and examined under a stereomicroscope at X3 and X4 magnification.

#### Percentage Mycorrhizal Colonization

Soybean roots were cut into pieces of 1 cm long and cleared in 10% KOH at 121 °C for 15 min in an autoclave (SA**-**300VL, Taiwan). Roots were covered with 2% HCl for 30 min after rinsing severally with distilled water. Root samples were subsequently covered with trypan blue solution (trypan blue solution consisted of 0.82 g trypan blue powder, 640 ml distilled water, 520 ml lactic acid and 480 ml glycerol) and autoclaved at 121 °C for 15 min. Root samples were then de-stained in 50% glycerol and examined under a stereo-microscope [16].

#### Below-Ground-Above-Ground Parameters

After harvesting soybean plants grown to maturity, data such as shoot height, shoot width, branch number, leaf number, taproot length, lateral root number, plant fresh weight, shoot dry weight and root dry weight were obtained according to the method described by Masciarelli et al. [28].

#### Above-Ground Yield Parameters

In this study, the following yield parameters were considered such as pod number, pod fresh weight, seed number, seed fresh weight, highest seed number per pod and seed dry weight. Seed fresh weight was determined by weighing seeds on a weighing machine. Seeds were oven-dried at 65 °C for 48 h and weighed on a weighing machine to obtain the dry weights.

### Statistical Analyses

In this experiment, the effects of rhizobia and water stress (drought) factors on soybean plant growth in the greenhouse were statistically analyzed. Test for homogeneity of data were done and data were normalized prior to using general linear model analysis of variance (ANOVA) to evaluate the impacts of rhizobia inoculants on soybean growth at three different water regimens or levels of 100, 70 and 40% using the following treatments which were replicated 8 times: Control_100% FC_, R1_100% FC_, R3_100% FC_, MY, R1MY _100% FC_, R3MY _100% FC_, R1+R3 _100% FC_, R1+R3MY _100% FC_. Control _70% FC_, R1 _70% FC_, R3 _70% FC_, MY _70% FC_, R1MY _70% FC_, R3MY _70% FC_, R1+R3 _70% FC_, R1+R3MY _70% FC_.Control _40% FC_, R1 _40% FC_, R3 _40% FC_, MY _40% FC_, R1MY _40% FC_, R3MY _40% FC_, R1+R3 _40% FC_, R1+R3MY _40% FC._

Differences between mean (post hoc test) was determined by Duncan’s multiple**-**range test (DMRT) [29] and differences were significant at *p* ≤ 0.05. DMRT was used in this study because is relatively more useful than other Post Hoc multiple comparison tests (such as least significant difference-LSD) when comparing larger pairs of means, particularly when the mean values are in a table.

## Results

### Soil Physicochemical Parameters

The soil that was used in this study contain sandy soil (70%) and silt (8%). The percentage of total nitrogen was low compared to 1.260 and 3.190% observed for organic carbon and organic matter, respectively (Table 1). Also, potassium (K) was 406 mg/kg while the soil pH which was 7.390 was within the pH range that supports bacterial growth.

**Table 1:** Physicochemical parameters of homogenized soil samples used for the greenhouse experiment

Parameter	Result
pH (H_2_O)	7.390
Fe (mg/kg)	1.950
Mn (mg/kg)	45.400
Organic carbon (%)	1.260
Organic matter (%)	3.190
Phosphorus	N/D
Potassium (mg/kg)	406.000
Magnesium (mg/kg)	639.00
Total nitrogen (%)	0.095
Sand (%)	70.000
Silt (%)	8.000
Clay (%)	22.000

### Leaf Relative Water Content

**Fig. 1:**
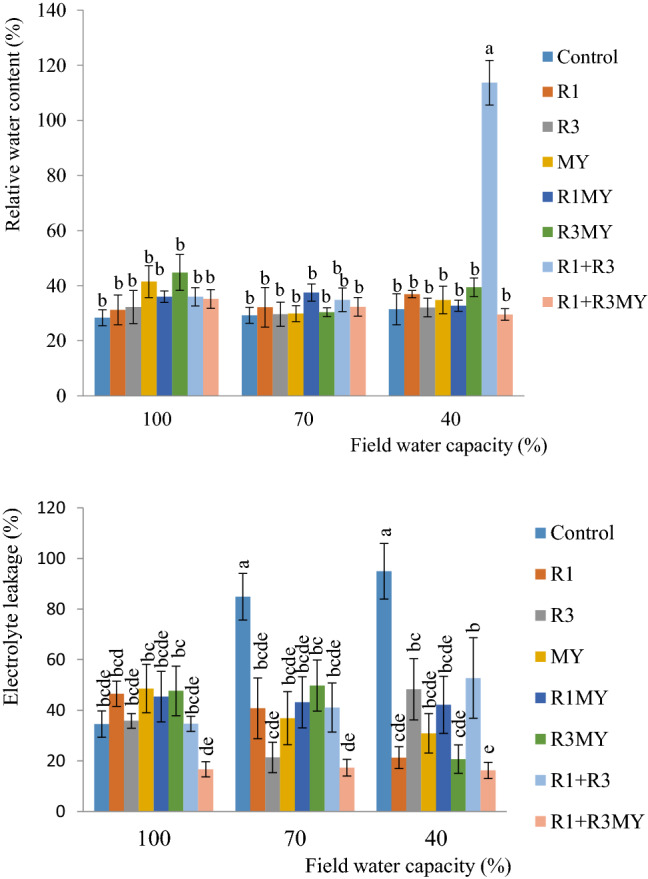
Relative water content and electrolytes of leaves of inoculated and non-inoculated soybean plants exposed to a 4-week period of drought stress. Control—non-inoculated treatment, R1—*Rhizobium* sp. strain R1, R3—*R. cellulosilyticum* strain R3, MY—mycorrhizal consortium, R1MY—*Rhizobium* sp. strain R1 and mycorrhizal fungal consortium, R3MY—*R. cellulosilyticum* strain R3 and mycorrhizal consortium, R1+R3—*Rhizobium* sp. strain R1 and *R. cellulosilyticum* strain R3, R1+R3MY—*Rhizobium* sp. strain R1, *R. cellulosilyticum* strain R3 and mycorrhizal consortium. Number of replicates (*n*) = 8. Data represent mean ± SE

Generally, the non**-**inoculated soybean plants were slightly affected (*p* > 0.05) with drought stresses (40% FC) compared to soybean plants inoculated with rhizobia and mycorrhizal fungi. In R1 + R3 treatment, soybean plants exposed to 40% FC maintained high relative water content in their leaves compared to the non**-**inoculated (control) plants (Fig 1).

### Leaf Electrolyte Leakage

As regards electrolyte leakage, with the exception of soybean plants amended with R1+R3MY, soybean plants inoculated with *R. cellulosilyticum* strain R3 (R3) showed a better result at 70% FC than other inoculated soybean plants. But at this level of water stress, significant increase (*p* < 0.05) was observed in non-inoculated treatment; the electrolyte leakage however decreased in 100% FC (Fig. 1). In all the water stress levels, the control treatments were the most negatively impacted particularly at 40% FC which showed electrolyte leakage of 94.9%.

### Proline Content of Soybean Leaves

**Fig. 2:**
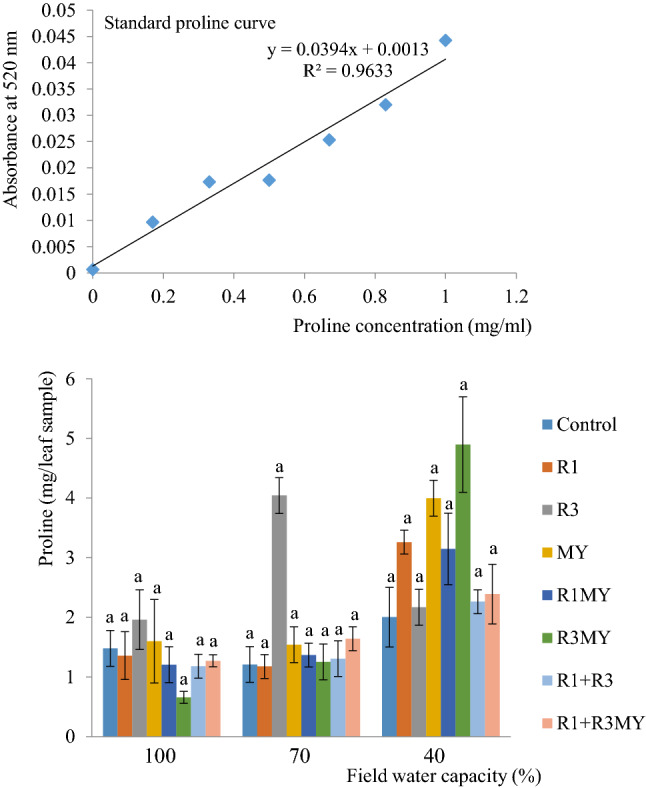
Proline accumulation in leaves of inoculated and non-inoculated soybean plants exposed to a 6-week period of drought stress. Control—non**-**inoculated treatment, R1—*Rhizobium* sp. strain R1, R3—*R. cellulosilyticum* strain R3, MY**—**mycorrhizal consortium, R1MY—*Rhizobium* sp. strain R1 and mycorrhizal fungal consortium, R3MY—*R. cellulosilyticum* strain R3 and mycorrhizal consortium, R1+R3—*Rhizobium* sp. strain R1 and *R. cellulosilyticum* strain R3, R1+R3MY—*Rhizobium* sp. strain R1, *R. cellulosilyticum* strain R3 and mycorrhizal consortium. Number of replicates (*n*) = 8. Data represent mean ± SE

Accumulation of proline in soybean leaves was highest in plants exposed to 40% water stress. Soybean plants dually inoculated with R3MY was found to accumulate the highest concentration of proline which was different (*p* > 0.05) from the non-inoculated (control) plants that produced the lowest amount of proline under 40% FC (Fig. 2). Also, all the microbially treated soybean plants produced more proline than the non-inoculated treatment under 40% FC. However, the non**-**inoculated soybean plants grown in 100% FC accumulated more proline (*p* > 0.05) than the non**-**inoculated soybean plants subjected to 70% FC.

### Chlorophyll Content of Soybean Leaves

**Fig. 3:**
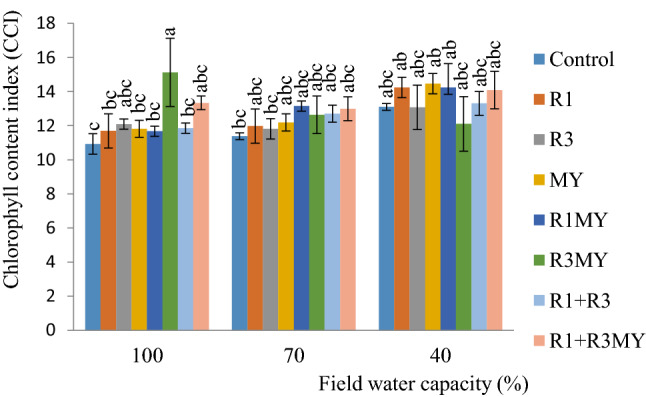
Chlorophyll content of leaves of inoculated and non**-**inoculated soybean plants exposed to a 3**-**week period of drought stress. Control—non**-**inoculated treatment, R1—*Rhizobium* sp. strain R1, R3—*R. cellulosilyticum* strain R3, MY**—**mycorrhizal consortium, R1MY—*Rhizobium* sp. strain R1 and mycorrhizal fungal consortium, R3MY—*R. cellulosilyticum* strain R3 and mycorrhizal consortium, R1+R3—*Rhizobium* sp. strain R1 and *R. cellulosilyticum* strain R3, R1+R3MY—*Rhizobium* sp. strain R1, *R. cellulosilyticum* strain R3 and mycorrhizal consortium. Number of replicates (*n*) = 8. Data represent mean ± SE

In soybean plants subjected to 40% water stress, R1, MY, R1MY and R1+R3MY inoculants were observed to increase the chlorophyll content of soybean leaves more than the control plants (exposed to 40, 70 and 100% FC) and their counterparts subjected to 70 and 100% water stress (Fig. 3). The non**-**inoculated (control) soybean plants grown in 70% FC showed the lowest chlorophyll content (11.375 CCI-chlorophyll content index) among all the plant treatments subjected to 70% water stress. At this water stress level, the greatest impact on chlorophyll content was detected in soybean plants dually amended with R1MY and R1 + R3MY

### Spore Number and Percentage Colonization of Mycorrhizal Fungi

**Fig. 4:**
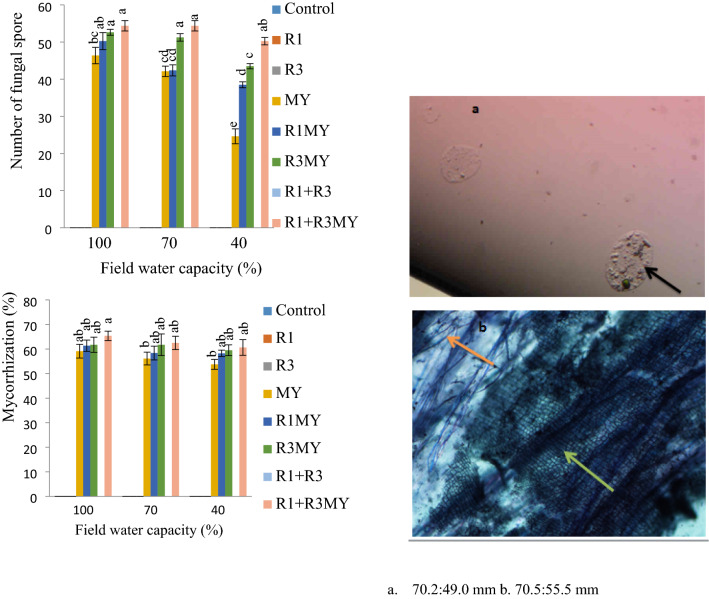
Spore number and percentage colonization level of mycorrhizal of inoculated and non-inoculated soybean plants exposed to a 10-week period of drought stress. Control—non-inoculated treatment, R1—*Rhizobium* sp. strain R1, R3—*R. cellulosilyticum* strain R3, MY—mycorrhizal consortium, R1MY—*Rhizobium* sp. strain R1 and mycorrhizal fungal consortium, R3MY—*R. cellulosilyticum* strain R3 and mycorrhizal consortium, R1+R3—*Rhizobium* sp. strain R1 and *R. cellulosilyticum* strain R3, R1+R3MY—*Rhizobium* sp. strain R1, *R. cellulosilyticum* strain R3 and mycorrhizal consortium. **a** Represents AMF spores (70.2:49.0 mm), **b** represents root colonized with AMF (70.5:55.5 mm). Black, green and orange arrows point towards fungal spores, arbuscule and hyphal, respectively. Number of replicates (*n*) = 8. Data represent mean ± SE

The treatment with the microbial combination R1+R3MY showed the highest number of spore in all the water levels (100, 70, 40% FC) while the lowest spore number was recorded for treatment solely inoculated with mycorrhizal consortium (MY). Control, R1, R3 and R1 + R3 treatments did not show presence of fungal spore (Fig. 4) since these treatments were not amended with mycorrhizal fungi (Fig. 4a).

Similar results were also observed for percentage (%) mycorrhization and the highest colonization levels were revealed in soybean plants co-inoculated with R1 + R3MY in all the water regimes. Mycorrhizal colonization (Fig. 4b) was observed to be more in the treatments co-inoculated with rhizobia and mycorrhizal fungi and coincidentally, colonization level in soybean plants inoculated with R1MY was approximately 58% in treatments subjected to 70 and 40% FC.

### Microbial Effects on Below-Ground and Above-Ground Parameters of Soybean Plants

Microbial (rhizobia and mycorrhizal) inoculation enhanced the growth of soybean plants virtually in all the water regimes compared to the non-inoculated (control) treatments (Table 2). We noticed that under different water regimes, inoculated soybean plants maintained higher below-ground-above-ground biomass (such as shoot height, shoot width, branch number, leaf number, taproot length, taproot width, lateral root number, plant fresh weight, shoot dry weight and root dry weight) compared to the non-inoculated plants (Table 2).

**Table 2:** Effects of rhizobia and mycorrhizal inoculation on below-ground-above-ground parameters of soybean plants exposed to a 16-week period of drought stress

Treatment	Shoot height (mm)	Shoot width (mm)	Branch number	Leaf number	Tap root length (mm)	Tap root width (mm)	Lateral root number	Plant fresh weight (g)	Shoot dry weight (g)	Root dry weight (g)
Control 40	171.4 ± 14.48^gh^	1.8 ± 0.16^i^	2.8 ± 0.33^gh^	4.6 ± 0.46^e^	49.9 ± 19.66^d^	1.3 ± 0.31^abcde^	12.9 ± 3.57^f^	1.03 ± 0.22^e^	0.3 ± 0.02^d^	0.1 ± 0.02^bc^
R1 40	195.9 ± 16.69^defgh^	3.1 ± 0.21^abc^	2.6 ± 0.63^h^	4.5 ± 1.27^e^	64.3 ± 14.12^cd^	1.1 ± 0.28^bcde^	14.3 ± 2.05^f^	1.2 ± 0.22^e^	0.4 ± 0.05^d^	0.1 ± 0.02^c^
R3 40	174.6 ± 6.21^fgh^	3.0 ± 0.19^abcd^	5.0 ± 0.46^cdefgh^	7.4 ± 0.82^e^	121.0 ± 20.29^abc^	1.5 ± 0.31^abcd^	13.9 ± 2.21^f^	1.3 ± 0.15^e^	0.6 ± 0.05^d^	0.1 ± 0.02^abc^
MY 40	185.9 ± 9.65^efgh^	1.9 ± 0.23^hi^	3.0 ± 0.42^gh^	8.6 ± 1.43^e^	123.1 ± 25.92^abc^	1.4 ± 0.17^abcd^	27.1 ± 4.36^abcde^	1.3 ± 0.18^e^	0.6 ± 0.07^d^	0.1 ± 0.02^abc^
R1MY 40	174.7 ± 14.59^fgh^	2.2 ± 0.19^fghi^	3.0 ± 0.78^gh^	8.9 ± 2.84^e^	91.6 ± 8.27^bcd^	1.3 ± 0.24^abcd^	29.6 ± 3.69^abcd^	1.5 ± 0.24^e^	0.6 ± 0.05^d^	0.2 ± 0.03^abc^
R3MY40	177.0 ± 12.72^fgh^	1.9 ± 0.13^hi^	3.9 ± 0.81^efgh^	10.8 ± 2.48^de^	97.3 ± 19.41^abcd^	0.9 ± 0.27^cde^	19.9 ± 3.94^def^	1.4 ± 0.23^e^	0.5 ± 0.05^d^	0.1 ± 0.03^abc^
R1+R3 40	182.3 ± 12.91^fgh^	2.0 ± 0.00^hi^	3.4 ± 0.37^fgh^	9.1 ± 1.13^e^	102.6 ± 13.57^abcd^	1.2 ± 0.21^bcde^	25.8 ± 2.58^bcde^	1.4 ± 0.15^e^	0.5 ± 0.04^d^	0.1 ± 0.02^abc^
R1+R3MY 40	159.0 ± 11.38^h^	3.1 ± 0.07^abc^	5.1 ± 0.63^cdefgh^	6.7 ± 1.78^e^	105.9 ± 12.70^abcd^	1.4 ± 0.14^abcd^	23.1 ± 1.42^abcdef^	1.3 ± 0.28^e^	0.5 ± 0.09^d^	0.2 ± 0.02^abc^
Control 70	206.0 ± 10.24^cdefgh^	1.9 ± 0.06^hi^	4.4 ± 0.43^defgh^	12.6 ± 1.32^de^	118.9 ± 21.31^abcd^	1.4 ± 0.18^abcd^	26.6 ± 3.13^abcde^	1.4 ± 0.13^e^	0.5 ± 0.12^d^	0.1 ± 0.01^abc^
R1 70	237.6 ± 8.45^bcd^	3.6 ± 0.18^ab^	8.1 ± 0.72^abc^	16.1 ± 2.17^cde^	97.4 ± 26.80^abcd^	1.3 ± 0.28^abcde^	22.4 ± 3.55^cdef^	2.0 ± 0.24^bcde^	0.7 ± 0.07^d^	0.2 ± 0.02^abc^
R3 70	209.9 ± 11.08^bcdefgh^	2.4 ± 0.26^defghi^	5.4 ± 0.75^cdefgh^	14.6 ± 3.04^cde^	134.6 ± 13.05^ab^	1.2 ± 0.12^abcde^	34.3 ± 2.14^ab^	1.5 ± 0.22^e^	0.6 ± 0.13^d^	0.1 ± 0.01^bc^
MY 70	240.3 ± 10.24^bcd^	2.3 ± 0.17^efghi^	5.4 ± 0.42^cdefgh^	17.1 ± 1.94^cde^	139.1 ± 21.31^ab^	1.6 ± 0.18^abc^	33.4 ± 3.13^abc^	1.7 ± 0.24^de^	0.6 ± 0.09^d^	0.1 ± 0.01^abc^
R1MY 70	209.6 ± 13.12^bcdefgh^	3.7 ± 0.14^a^	8.1 ± 0.67^abc^	19.5 ± 3.57^cde^	113.1 ± 11.15^abcd^	1.4 ± 0.11^abcd^	27.1 ± 1.33 ^abcde^	4.2 ± 0.98^e^	1.1 ± 0.26^dc^	0.2 ± 0.26^abc^
R3MY 70	222.9 ± 12.35^bcdefg^	2.6 ± 0.25^cdefgh^	5.4 ± 0.49^cdefgh^	16.1 ± 2.35^cde^	168.0 ± 12.50^ab^	0.5 ± 0.07^e^	16.6 ± 0.88^ef^	1.4 ± 0.23^e^	0.8 ± 0.14^d^	0.2 ± 0.02^abc^
R1+R3 70	225.6 ± 10.17^bcdef^	2.1 ± 0.29^ghi^	6.3 ± 1.56^bcdef^	15.9 ± 2.94^cde^	142.0 ± 27.94^ab^	1.3 ± 0.21^abcde^	27.4 ± 3.16^abcde^	1.9 ± 0.55^cde^	0.7 ± 0.14^d^	0.2 ± 0.07^abc^
R1+R3MY70	259.8 ± 17.91^ab^	2.3 ± 0.16^defghi^	5.0 ± 0.42^cdefgh^	17.5 ± 3.01^cde^	143.3 ± 23.14^ab^	1.6 ± 0.171^abcd^	30.4 ± 2.95^abcd^	1.9 ± 0.34^cde^	0.7 ± 0.16^d^	0.2 ± 0.02^abc^
Control 100	214.4 ± 33.71^bcdefgh^	2.1 ± 0.21^ghi^	6.1 ± 0.85^bcdefg^	18.5 ± 5.15^cde^	89.75 ± 16.75^bcd^	0.8 ± 0.13^de^	2.0 ± 0.57^ef^	0.8 ± 0.25^f^	0.8 ± 0.25^d^	0.2 ± 0.03^abc^
R1 100	240.4 ± 20.33^bcd^	2.8 ± 0.23^cdefg^	9.1 ± 1.75^ab^	37.5 ± 10.92^ab^	107.5 ± 20.98^abcd^	1.4 ± 0.36^abcd^	33.0 ± 4.95^abc^	4.9 ± 1.58^a^	2.3 ± 0.81^ab^	0.3 ± 0.06^ab^
R3 100	247.1 ± 21.39^abcd^	2.3 ± 0.29^fghi^	6.3 ± 1.72^bcde^	21.9 ± 9.73^bcde^	96.0 ± 30.16^abcd^	1.2 ± 0.39^abcde^	29.3 ± 5.35^abcd^	3.6 ± 1.95^abcde^	1.3 ± 0.78^bcd^	0.3 ± 0.12^abc^
MY 100	291.3 ± 13.19^a^	2.9 ± 0.35^bcdef^	9.5 ± 0.93^a^	47.8 ± 8.99^a^	155.9 ± 15.64^ab^	1.8 ± 0.25^ab^	37.5 ± 3.53^a^	4.4 ± 1.19^ab^	2.5 ± 0.73^a^	0.3 ± 0.06^a^
R1MY 100	260.9 ± 20.42^ab^	2.8 ± 0.31^cdefg^	8.8 ± 1.66^ab^	39.0 ± 13.54^ab^	166.9 ± 19.76^a^	1.3 ± 0.15^abcde^	31.1 ± 4.14^abcd^	3.4 ± 0.9^abcde^	2.0 ± 0.71^abc^	0.3 ± 0.06^abc^
R3MY 100	236.5 ± 15.66^bcde^	3.0 ± 0.41^abcde^	7.4 ± 1.13^abcd^	28.5 ± 9.75^bcd^	94.4 ± 17.27^bcd^	1.9 ± 0.17^a^	30.9 ± 4.16^abcd^	4.3 ± 1.29^abc^	1.3 ± 0.42^d^	0.1 ± 0.05^abc^
R1+R3 100	251.8 ± 19.56^abc^	3.7 ± 0.29^a^	6.8 ± 0.90^abcde^	22.1 ± 4.79^bcde^	149.8 ± 30.93^ab^	0.9 ± 0.12^cde^	29.8 ± 4.69^abcd^	2.8 ± 0.62^abcde^	0.9 ± 0.30^dc^	0.2 ± 0.03^abc^
R1+R3MY 100	246.8 ± 13.14^abcd^	3.8 ± 0.31^a^	8.1 ± 0.58^abc^	33.0 ± 4.72^abc^	128.9 ± 16.2^abc^	0.8 ± 0.12^cde^	25.5 ± 1.94^bcde^	4.1 ± 0.51^abcd^	1.3 ± 0.18^bcd^	0.2 ± 0.04^abc^

Control—non—inoculated treatment, R—*Rhizobium* sp. strain R1, R3—*R. cellulosilyticum* strain R3, MY—Mycorrhizal consortium, R1MY—*Rhizobium* sp. strain R1 and mycorrhizal fungal consortium, R3MY—*R. cellulosilyticum* strain R3 and mycorrhizal consortium, R1+R3—*Rhizobium* sp. strain R1 and *R. cellulosilyticum* strain R3, R1+R3MY—*Rhizobium* sp. strain R1, *R. cellulosilyticum* strain R3 and mycorrhizal consortium, mm—millimeter, g—gram. Data represent mean ± SE. According to Duncan’s multiple-range test (*n* = 8), values that have common letters are not different significantly (*p* > 0.05)

### Microbial Effects on Soybean Yield

Irrespective of the water treatment, rhizobial and mycorrhizal inoculation increased soybean yield components such as pod number, pod fresh weight, seed number, seed fresh weight, highest seed number per pod and seed dry weight. For 40% FC, R1 + R3MY inoculum had the greatest impact on pod number, seed number, seed fresh weight, highest seed number per pod and seed dry weight (Table 3). But R1MY and R1 + R3 were observed to have the greatest effects on pod fresh weight with a mean weight of 0.16 g each at this level of water stress. However, the non-inoculated plants and plants that were singly inoculated with R1 and R3 did not produce seeds even though they had pods. Significant effects (*p* < 0.05) of R1 + R3MY inoculation were not only observed on seed number, seed fresh weight and seed dry weight but also on pod number and highest number per pod compared to other microbial and non-microbial or control treatments subjected to the 40% FC.

**Table 3:** Effects of rhizobia and mycorrhizal inoculation on the yield components of soybean plants exposed to a 16-week period of drought stress

Treatment	Pod number	Pod fresh weight (g)	Seed number	Seed fresh weight (g)	Highest seed number per pod	Seed dry weight (g)
Control 40	0.50 ± 0.33^de^	0.01 ± 0.00^d^	0.00 ± 0.00^d^	0.00 ± 0.00^c^	0.00 ± 0.00^f^	0.00 ± 0.00^b^
R1 40	0.25 ± 0.25^e^	0.03 ± 0.03^d^	0.00 ± 0.00^d^	0.00 ± 0.00^c^	0.00 ± 0.00^f^	0.00 ± 0.00^b^
R3 40	0.13 ± 0.44^e^	0.01 ± 0.13^d^	0.00 ± 0.00^d^	0.00 ± 0.00^c^	0.00 ± 0.00^f^	0.00 ± 0.00^b^
MY 40	0.88 ± 0.38^bcde^	0.13 ± 0.20^cd^	0.510 ± 0.59^d^	0.04 ± 0.09^c^	0.75 ± 0.38^bcdef^	0.03 ± 0.03^b^
R1MY 40	0.50 ± 0.87^de^	0.16 ± 0.61^cd^	0.63 ± 1.36^d^	0.05 ± 0.31^c^	0.50 ± 0.31^def^	0.01 ± 0.12^b^
R3MY 40	0.38 ± 0.35^de^	0.05 ± 0.33^d^	0.25 ± 0.74^d^	0.01 ± 0.07^c^	0.25 ± 0.41^ef^	0.02 ± 0.02^b^
R1+R3 40	0.25 ± 0.25^e^	0.16 ± 0.11^cd^	0.75 ± 0.49^d^	0.07 ± 0.05c	0.50 ± 0.33^def^	0.03 ± 0.02^b^
R1+R3MY 40	0.57 ± 0.43^bcde^	0.10 ± 0.08^cd^	1.14 ± 0.73^bcd^	0.10 ± 0.07^bc^	1.14 ± 0.73^abcd^	0.093 ± 0.08^ab^
Control 70	1.12 ± 0.35^bcde^	0.12 ± 0.04^cd^	0.50 ± 0.19^d^	0.05 ± 0.19^c^	0.50 ± 0.19^def^	0.01 ± 0.00^b^
R1 70	1.25 ± 0.37^bcde^	0.27 ± 0.09^cd^	0.88 ± 0.39^d^	0.06 ± 0.03^c^	0.75 ± 0.31^bcdef^	0.04 ± 0.02^b^
R3 70	0.88 ± 0.44^bcde^	0.25 ± 0.13^cd^	1.13 ± 0.58^dc^	0.11 ± 0.07^bc^	0.75 ± 0.37^bcdef^	0.03 ± 0.02^b^
MY 70	1.00 ± 0.38^bcde^	0.53 ± 0.21^bcd^	1.50 ± 0.59^bcd^	0.19 ± 0.09^bc^	1.00 ± 0.38^abcdef^	0.06 ± 0.03^b^
R1MY70	2.50 ± 0.87^abcde^	1.17 ± 0.61^abc^	2.88 ± 1.35^abcd^	0.50 ± 0.31^abc^	1.25 ± 0.31^abcde^	0.19 ± 0.12^ab^
R3MY 70	1.13 ± 0.35^bcde^	0.95 ± 0.33^abcd^	1.13 ± 0.74^dc^	0.07 ± 0.07^c^	0.63 ± 0.42^cdef^	0.03 ± 0.02^b^
R1+R3 70	2.13 ± 0.58^abcde^	0.65 ± 0.24^bcd^	3.00 ± 0.96^abcd^	0.28 ± 0.11^abc^	1.25 ± 0.37^abcde^	0.14 ± 0.05^ab^
R1+R3MY 70	1.88 ± 0.39^abcde^	0.56 ± 0.25^bcd^	2.25 ± 0.77^bcd^	0.26 ± 0.15^bc^	1.25 ± 0.31^abcde^	0.08 ± 0.05^b^
Control 100	0.17 ± 0.11^cde^	0.17 ± 0.11^cd^	0.50 ± 0.27^d^	0.03 ± 0.02^c^	0.50 ± 0.28^def^	0.01 ± 0.01^b^
R1 100	4.38 ± 1.99^a^	1.82 ± 0.89^a^	6.25 ± 2.96^a^	0.78 ± 0.41^a^	1.75 ± 0.49^abc^	0.23 ± 0.12^ab^
R3 100	2.75 ± 1.19^abcde^	0.79 ± 0.37^abcd^	2.63 ± 1.05^bcd^	0.29 ± 0.13^abc^	1.13 ± 0.39^abcdef^	0.08 ± 0.04^b^
MY 100	3.38 ± 1.71^abc^	0.99 ± 0.53^abcd^	2.51 ± 1.54^abcd^	0.27 ± 0.19^abc^	2.88 ± 0.39^abcdef^	0.37 ± 0.07^ab^
R1MY 100	3.13 ± 0.58^abcd^	0.76 ± 0.21^bcd^	2.25 ± 0.90^bcd^	0.22 ± 0.09^bc^	0.88 ± 0.29^abcdef^	0.06 ± 0.03^b^
R3MY100	3.50 ± 1.28^ab^	1.17 ± 0.46^abc^	5.00 ± 2.33^ab^	0.61 ± 0.32^ab^	2.00 ± 0.38^a^	0.35 ± 0.28^a^
R1+R3 100	2.88 ± 0.95^abcde^	0.80 ± 0.24^abcd^	2.88 ± 0.74^abcd^	0.31 ± 0.09^abc^	1.75 ± 0.31^abc^	0.09 ± 0.03^ab^
R1+R3MY100	4.13 ± 1.16^a^	1.38 ± 0.46^ab^	4.63 ± 1.31^abc^	0.62 ± 0.28^ab^	1.88 ± 0.48^ab^	0.23 ± 0.12^ab^

Control—non-inoculated treatment, R1—*Rhizobium* sp. strain R1, R3—*R. cellulosilyticum* strain R3, MY**—**Mycorrhizal consortium, R1MY—*Rhizobium* sp. strain R1 and mycorrhizal fungal consortium, R3MY—*R. cellulosilyticum* strain R3 and mycorrhizal consortium, R1+R3—*Rhizobium* sp. strain R1 and *R. cellulosilyticum* strain R3, R1+R3MY—*Rhizobium* sp. strain R1, *R. cellulosilyticum* strain R3 and mycorrhizal consortium, 40—40% field water capacity, 70—70% field water capacity, 100—100% field water capacity, g**—**gram. Data represent mean ± SE. According to Duncan’s multiple-range test (*n* = 8), values that have common letters are not different significantly (*p* > 0.05)

On the other hand, we observed that the effects of R1+R3MY treatment decreased at 70% FC, but the decrease was only significant for highest seed number per pod and seed dry weight (Table 3). At this level of water stress, significant impacts (*p* < 0.05) of R1MY inoculation were observed on pod number, pod fresh weight, seed number and seed dry weight (Table 3). Unlike in 40% FC, the control, R1 and R3 treatments produced seeds at 70% FC. Overall, 70% water stress negatively affected the control soybean plants compared to the microbially amended plants (Table 3). In particular, the mean pod fresh weight, seed number, seed fresh weight, highest seed number per pod and seed dry weight observed in the control treatment corresponds to 0.12 g, 0.5, 0.05 g, 0.05 and 0.01 g but only the highest seed number per pod was significantly less than all inoculated treatments while others were not significantly less (*p* > 0.05) than some of the inoculated treatments (Table 3).

A similar pattern was noticed in inoculated and non-inoculated plants grown in 100% water level. R1 treatment produced more seed with the greatest pod and seed fresh weight. Generally, under this water regime, the non-inoculated plants were less productive compared to the other treatments as depicted by the yield components measured (Table 3).

## Discussion

In this study, physiological, biochemical and morphological parameters connected to drought tolerance in soybean plants amended with rhizobia and mycorrhizal consortium under water stress conditions were investigated.

Our results indicated that the singly and dually inoculated soybean plants showed different levels of percentage relative water content (% RWC) under 40, 70 and 100% FC. However, water stress reduced the RWC in non-inoculated soybean plants exposed to both water stressed treatments and plants inoculated with R1 + R3MY grown in 40% FC (Fig. 1). Overall, the current study unveiled that microbial inoculations enhanced RWC in soybean plant leaves under water stress.

RWC is used to evaluate water level balance in plants and it is a dependable strategy by which the level of osmotic stress is determined [23]. Indeed, RWC helps to determine the level of water in plants which is pointer to the metabolic state in plant tissues. Under water stress, RWC is usually considered an excellent indicator of drought stress tolerance in different plants [16]. Thus, in this study, MY treatment was able to increase RWC from 29.8 (in 70% FC) to 34.8% (in 40% FC). This confirms the reports that mycorrhizal fungi increased leaf RWC [30, 31]. In addition, some single and dual inoculations similarly led to increase in RWC and this increase was pronounced in plants treated with R1 + R3. The mechanisms associated with this increase in RWC by microbial isolates is linked to as reported by Zarik et al. [23] “stomatal regulation through hormonal signals [31]; higher stomatal conductance and transpiration fluxes [32]; indirect effect of improved phosphate and other nutrient uptake [33]; greater osmotic adjustment [34] and/or higher root hydraulic conductivity [13]” than non-inoculated (control) plants.

Plant responses to water stress have also been evaluated based on physiological values derived from electrolyte leakage [26] which in the present study were lowest particularly in soybean plants dually inoculated with rhizobial spp. and mycorrhizal consortium (R1 + R3MY) and highest in the non-inoculated plants (Fig. 1). The low level of electrolyte leakage is a pointer to “cell membranes stability” and drought stress tolerance [35]. Cell membrane is very crucial in monitoring the efficacy of plant root-microbial interactions [36]. Most of the microbial inocula used in this study reduced plant membrane damaged in drought stressed soybean plants since they showed relatively low % electrolyte leakage compared to the non-inoculated plants. This result is in agreement with the results obtained by Ortiz et al. [26] who reported low electrolyte leakage values in *Trifolium repens* amended with selected (PGPR) and AMF in arid soil environment.

The amino acid proline is an essential ‘osmo-protectant osmolyte’ produced by plants to improve osmosis and prevent water loss [23, 37]. The proline concentration in leaves was lower in non-inoculated soybean plants than in rhizobial and mycorrhizal inoculated soybean plants under severe drought (Fig. 2). Under this drought condition, significant accumulation of proline was observed in the leaves of soybean plants treated with the dual inoculum R3MY (Fig. 2). This finding corresponds to the result of Ortiz et al. [26] who reported significant proline accumulation in some plant treatments dually inoculated with microbial species leading to a reduction in the osmotic potential of the plant cells and consequently enhanced uptake of water to ‘maintain osmotic balance’ [38]. Also, the concentration of proline accumulated by soybean plants treated with MY under severe drought condition was approximately 4 µmol/g dry weight which was comparatively higher than that of the non-mycorrhized treatments (control, R1, R3, R1 + R3). This result disagrees with the conclusions of Zarik et al. [23]; Manoharan et al. [39] and Wu and Xia [34] who observed less proline content in the leaves of mycorrhized *Cupressus atlantica*, vagiegata and orange plants respectively than in non-mycorrhized plants grown under moderate and severe drought stresses. Furthermore, under moderate drought stress condition, soybean plants treated with R1 + R3MY were shown to accumulate the highest level of proline while the lowest level was found in R1 treatment. Contrary results were found in the well-watered plant treatments where the highest and lowest proline concentrations were produced by plants inoculated with R3 and R3MY, respectively.

Surprisingly, lower chlorophyll content was observed in virtually all soybean plants grown in 100% FC and increased with increasing drought stress (Fig. 3). Overall, R3MY treatment and non-inoculated plants grown in 100% FC showed the highest (15.2 CCI) and lowest (10.9 CCI) chlorophyll content, respectively. Chlorophyll is an important component that plays a vital role in the process of photosynthesis in plants [40]. Considering the different water levels, chlorophyll content negatively correlated with below-ground-above-ground productivity, implying that as the drought stress increased, chlorophyll content increased and plant productivity decreased.

Furthermore, we found that the number of mycorrhizal spore (Fig. 4) and % mycorrhization in this study were dominant in soybean treated with R1 + R3MY under 40, 70 and 100% FC. Also, soybean root colonization or % mycorrhization (Fig. 4) was positively correlated with RWC (Fig. 1) and seed number of soybean plants inoculated with MY, R3MY and R1 + R3MY (Table 3) indicating enhancement of plant water status and productivity. Perhaps, mycorrhizal fungi may have contributed to drought avoidance capacity of these treatments, thus resulting in water stress alleviation in soybean plants. The increase in seed number and other morphological components in soybean plants inoculated with MY, R1MY, R3MY, R1 + R3MY may partly be ascribed to mycorrhizal colonization (Fig. 4b) and RWC (especially for MY, R1MY and R3MY treatments at 40% FC), signifying that plant water content was efficiently enhanced by this plant-microbial interactions resulting in enhanced productivity. This finding agrees with the results of Aliasgharzad et al. [19] who reported that an increase in % mycorrhization led to an increase in RWC in soybean plants inoculated with both *Bradyrhizobium japonicum* and *Glomus etunicatum.*

It was reported in our previous work [41] that below-ground interactions between plants and rhizobacteria/AMF can trigger responses that may affect above-ground plant components. But in the present study, it became evident that below-ground interactions among rhizobia, mycorrhizal fungi and soybean plants under stressed condition improved the productivity of most of the above-ground components such as shoot height, shoot width, branch number, leaf number, shoot dry weight, pod number, pod fresh weight, seed number, seed fresh weight, highest seed number per pod and seed dry weight (Tables 2 and 3).

At 40% FC, significant promotion of soybean plant growth under severe drought stress by R1 + R3MY inoculation depicted by increased in shoot width and branch number (Table 2) could be as a result of the synergistic interaction between the microbial species and mutualistic interaction between the inoculum and soybean plant roots [41]. This treatment in addition to the dual inoculum R1MY greatly affected root dry weight, although the effect was only significantly different (*p* < 0.05) from plants inoculated with R1. Similarly, under severe drought stress, taproot length was greatly affected by R3 inoculum and this result somewhat supported the outcome of our previous work (data not shown) in which R3 significantly affected soybean seedling root fresh and dry weight and number of lateral root in a growth chamber experiment where PEG was used as a drought factor. But in this study, under severe drought stressed condition, R3 treatments produced lateral roots with a mean value of 13.9 which was less than that produced by other microbial treatments but higher than that of non-inoculated plants (Table 2).

On the contrary, R3 treatments produced the highest (34.3) number of lateral roots under moderate drought stress (70% FC) which further supports our previous PEG experiment results (Table 2). Like in severe drought condition, R1 + R3MY inoculum also enhanced soybean growth under moderate drought stress since it had the greatest effect on soybean shoot height under this condition (Table 2). Below-ground taproot length received the greatest impacts from both R1 + R3MY and MY inocula compared to other treatments. Under this condition, we observed that below-ground synergistic interaction between *Rhizobium* sp. strain R1 and mycorrhizal consortium (RIMY) outstandingly impacted soybean shoot width, branch number, leaf number and shoot number.

For the well-watered treatments, it was found that MY glaringly enhanced soybean plant shoot height with a mean value of 291.3 mm (Table 2) and the non-inoculated plants showed the poorest shoot development as they presented the lowest (214 and 2.1 mm) shoot height and shoot width, respectively. Thus, based on the shoot height and shoot width evaluation, the non-inoculated or control plants produced soybean plants with the lowest shoot biomass in all the water regimes (Table 2).

One of the indicators of sustainable agriculture and/or food security is increase in agricultural produce [18, 42]. Our present study has shown that combination of rhizobia and mycorrhizal fungal consortium resulted in increased yield (soybean seed number) under severe and moderate stressed conditions in a controlled environment (Table 3). Particularly, R1 + R3MY treatments significantly (*p* < 0.05) produced more seeds in 40% FC than other treatments (Table 3). The non-inoculated (control) plants and plants amended with R1 and R3 did not produced seeds under this condition probably because the absence of PGPR (in the control plants) and single inoculation (in R1 and R3 treated plants) could not ameliorate the detrimental effects of drought stress to the extent of empowering the plants to produced seeds even though these treatments produced pods. It was reported in our previous studies [43, 44] that abiotic stresses such as drought, heat and salinity are part of the factors that militate against crop growth, development and yield. The impacts of these abiotic stresses may be abated by below-ground plant-microbial interactions [41]. Tripartite mutualistic interactions with AMF fungi and *Rhizobium* species [41] is therefore important for increased productivity in soybean [18] and the impacts of co-inoculation with mycorrhizal fungi and rhizobia on soybean plants need further research.

Also, co-inoculation with mycorrhizal consortium and rhizobia (R1MY) significantly enhanced soybean yield as indicated by pod number, pod fresh weight, seed number and seed fresh weight in the 70% FC but not in the 100% FC (Table 3). These outcomes further showed that there was a synergistic impact between mycorrhizal consortium and rhizobia on soybean yield in this investigation and that this impact ‘might’ be dependent on water status. However, previous study by Wang et al. [45] indicated that such synergistic impact can be link to nutrient status. It is generally believed that mycorrhizal fungi principally benefit plants grown in soil environments where phosphorus is likely to hinder plant productivity by increasing the volume of soil penetrated by mycorrhizal hyphae compared to that of root hairs of non-mycorrhizal plants [43, 45] and in the current study, there was no addition of inorganic phosphorus and nitrogen to the soil and the natural soil total nitrogen was slightly low (00.095%-Table 1) compared to that (0.22%) reported by Ortiz et al. [26]. This low nutrient level of soil could be another reason (In addition to water status) for the synergistic impacts of dual inoculation with mycorrhizal consortium and rhizobia in this study. The universal consensus is that mycorrhizal fungi enhance phosphorus nutrient uptake in legumes which eventually improves plant development and nitrogen fixation [15, 16] and according to Xavier and Germida [46], dual inoculation with rhizobia species and compatible AMF can intensely improve pea growth and nutrient uptake. On the basis of this, we observed that the rhizobia and mycorrhizal consortium used in the present study enhanced soybean plants tolerance to drought and improved their productivity. Therefore, the fungal consortium is compatible with our rhizobia species and soybean cultivar, which may have prospect for agronomic application.
